# Connecting the Dots: The Cerebral Lymphatic System as a Bridge Between the Central Nervous System and Peripheral System in Health and Disease

**DOI:** 10.14336/AD.2023.0516

**Published:** 2024-02-01

**Authors:** Hongxiang Zhao, Meiyan Sun, Yue Zhang, Wenwen Kong, Lulu Fan, Kaifang Wang, Qing Xu, Baiyan Chen, Jianxin Dong, Yanan Shi, Zhengyan Wang, ShiQi Wang, Xiaoli Zhuang, Qi Li, Feihong Lin, Xinyu Yao, WenBo Zhang, Chang Kong, Rui Zhang, Dayun Feng, Xiaoyong Zhao

**Affiliations:** ^1^Shandong Cancer Hospital and Institute, Shandong First Medical University and Shandong Academy of Medical Sciences, Jinan, Shandong, China.; ^2^Department of Anesthesiology, Affiliated Hospital of Weifang Medical University, Weifang, China.; ^3^Shandong Provincial Medicine and Health Key Laboratory of Clinical Anesthesia, School of Anesthesiology, Weifang Medical University, Weifang, China.; ^4^Department of neurosurgery, Tangdu hospital, Fourth Military Medical University, Xi'an, China.; ^5^Department of Anesthesiology, Affiliated Hangzhou First People's Hospital, Zhejiang University School of Medicine, Hangzhou, China.; ^6^Department of Anesthesiology, Beijing Anzhen Hospital, Capital Medical University, Beijing, China.; ^7^Department of Anesthesiology, Ninth People's Hospital, Shanghai Jiaotong University School of Medicine, Shanghai, China.; ^8^Department of Anesthesiology, Shanghai Pulmonary Hospital, School of Medicine, Tongji University, Shanghai, China.; ^9^Department of Anesthesiology, The First Affiliated Hospital of Wenzhou Medical University, Wenzhou, China.; ^10^Department of Neurosurgery, The Children’s Hospital of Zhejiang University School of Medicine, National Clinical Research Center for Child Health, Hangzhou, China.; ^11^Department of Anesthesiology and Critical Care Medicine, Tianjin Nankai Hospital, Tianjin Medical University, Tianjin, China.

**Keywords:** Cerebral lymphatic system, peripheral system, meningeal lymphatic vessels, glymphatic system, gastrointestinal tract, liver, kidney

## Abstract

As a recently discovered waste removal system in the brain, cerebral lymphatic system is thought to play an important role in regulating the homeostasis of the central nervous system. Currently, more and more attention is being focused on the cerebral lymphatic system. Further understanding of the structural and functional characteristics of cerebral lymphatic system is essential to better understand the pathogenesis of diseases and to explore therapeutic approaches. In this review, we summarize the structural components and functional characteristics of cerebral lymphatic system. More importantly, it is closely associated with peripheral system diseases in the gastrointestinal tract, liver, and kidney. However, there is still a gap in the study of the cerebral lymphatic system. However, we believe that it is a critical mediator of the interactions between the central nervous system and the peripheral system.

## 1. Introduction

For a long time, the brain was thought to lack the presence of the lymphatic system. As a result, it may be in an immune-exempt state. So how exactly does the brain maintain its normal state of physiological function? A further understanding of the anatomy of the brain may be the key to solving this question. Numerous studies have shown that there may be a special lymphatic system in the brain - the cerebral lymphatic system. Paolo Mascagni first discovered the existence of a special structure, the meningeal lymphatic vessels in the brain, as early as the end of the 18th century [1]. However, due to the existence of paradigmatic thought and the limitation of science and technology, this theory has not been widely accepted by academia. Finally, in 2015, the existence of meningeal lymphatic vessels was confirmed and became an accepted concept [2]. At the same time, in 2012, Maiken Nedergaard and his team introduced the concept of the glymphatic system and corroborated the existence of this lymphatic structure in mouse models. Over the following years, the existence of this structure has also been demonstrated in the human brain [3-5]. Our understanding of the cerebral lymphatic system continues to improve with the development of imaging techniques. This has provided conclusive evidence for its existence and revolutionised our understanding of the hydrodynamics of fluid flow in the brain and of brain immunity. In 2023, the study of the cerebral lymphatic system entered a new historical phase with the discovery of the subarachnoidal lymphatic-like membrane [6]. As a novel concept, the cerebral lymphatic system has successfully changed our traditional perception of the anatomy of the brain and its functions [2, 3, 7, 8]. Several studies have demonstrated that it has similar functions to the peripheral lymphatic system, such as the efficient removal of metabolic waste and immune monitoring. At the same time, however, the unique structure of the cerebral lymphatic system and its drainage patterns break down our traditional perception of the lymphatic system. For example, the glymphatic system lacks the walls of typical lymphatic vessels and the space within its lumen is largely occupied by blood vessels. These findings have given us new insights into the pathogenesis of a variety of central nervous system diseases, such as brain tumour, cerebral haemorrhage, hydrocephalus, stroke and neurodegenerative diseases [9-12]. More interestingly, the cerebral lymphatic system has been shown to play an essential role in the interaction of the central nervous system with peripheral system such as the liver, gastrointestinal tract and kidney [13-16]. At the same time, it also performs the function of transporting brain antigens and immune cells. This provides a channel for immune-related communication between the central nervous system and the peripheral system [11, 17]. Currently, two main directions have been derived for the study of the cerebral lymphatic system: the meningeal lymphatics and the glymphatic system, which focus on the functional characteristics of the cerebral lymphatic system in physiological and pathological conditions [18-21]. In this paper, we review the current literature on the structure, physiological functions and properties of the cerebral lymphatic system and present a theoretical framework based on the structure and function of the cerebral lymphatic system as a whole. More importantly, the role of the cerebral lymphatic system as a bridge between the central nervous system and the peripheral system was reviewed and summarised, which may provide ideas for future research in this field and further improve the body of knowledge on the cerebral lymphatic system.

## 2. Structural components of the cerebral lymphatic system

Brain parenchyma produces various metabolic wastes during physiological or pathological processes. If these metabolic wastes are not removed effectively and in a timely manner, abnormalities in brain function may be induced. This can ultimately lead to the development and progression of a range of brain diseases [22]. Various studies have shown that the cerebral lymphatic system plays an important role in the transport and elimination of metabolic wastes in the central nervous system (CNS) [11, 23, 24]. The cerebral lymphatic system can exert its drainage role through two pathways - the glymphatic system pathway and the cerebrospinal fluid drainage pathway. The former consists mainly of the perivascular space (Virchow-Robin space) and the cerebrospinal fluid-interstitial fluid flow in the brain parenchyma. The latter consists mainly of meningeal lymphatics and olfactory/ cervical lymphatic pathways [22]. However, the precise anatomical pathways of the cerebral lymphatic system drainage are still unclear. According to the current knowledge of the cerebral lymphatic system, it consists mainly of the meningeal lymphatic vessels and the glymphatic system. Some metabolites in the central nervous system first enter the meningeal lymphatics from the deep brain through the glymphatic system and then enter the peripheral lymphatic system [2]. Substance exchange is the main function of the cerebral lymphatic system. Its purpose is to drain metabolic wastes accumulated in the brain parenchyma and to import nutrients. The meningeal lymphatic vessels and the glymphatic system are closely related but have subtle functional differences, and they function together by pairing each other. Further understanding and study of the anatomy of the cerebral lymphatic system may provide new ideas for the treatment of central nervous system diseases. We will present them separately next.

### 2.1 Meningeal lymphatic vessels

Meningeal lymphatic vessels (MLVs) are the main route for the removal of intracranial cerebrospinal fluid (CSF) and interstitial fluid (ISF) to the deep cervical lymph nodes (dCLN) outside the skull. To some extent, it can be considered as a bridge between intracranial and extracranial areas [25]. Meningeal lymphatics have typical characteristics of lymphatic vessels, mainly due to the presence of typical fully differentiated lymphatic endothelial cells, which are PROX1^+^/VEGFR3^+^/ LYVE1^+^/PDPN^+^/CCL21^+^/PECAM1^low^. The meningeal lymphatics are mainly distributed along the superior sagittal sinus, surrounding the transverse sinus, sigmoid sinus, posterior cranial fossa veins and middle meningeal artery. The meningeal lymphatics are sparsely distributed in the cranial vault and densely distributed in the cranial base. At the base of the skull, the meningeal lymphatics follow the distal cranial nerves, including the optic nerve, trigeminal nerve, glossopharyngeal nerve, paramedian nerve, and vagus nerve. Subsequently, it leaves the skull along with the cranial nerves through the neuroforamen of the skull base and eventually enters the deep cervical lymph nodes [7]. In addition to this, the meningeal lymphatic vessels are present in the sieve plate for distribution. It is able to leave the skull here and eventually enters the nasal mucosa [26].

**Figure 1.**
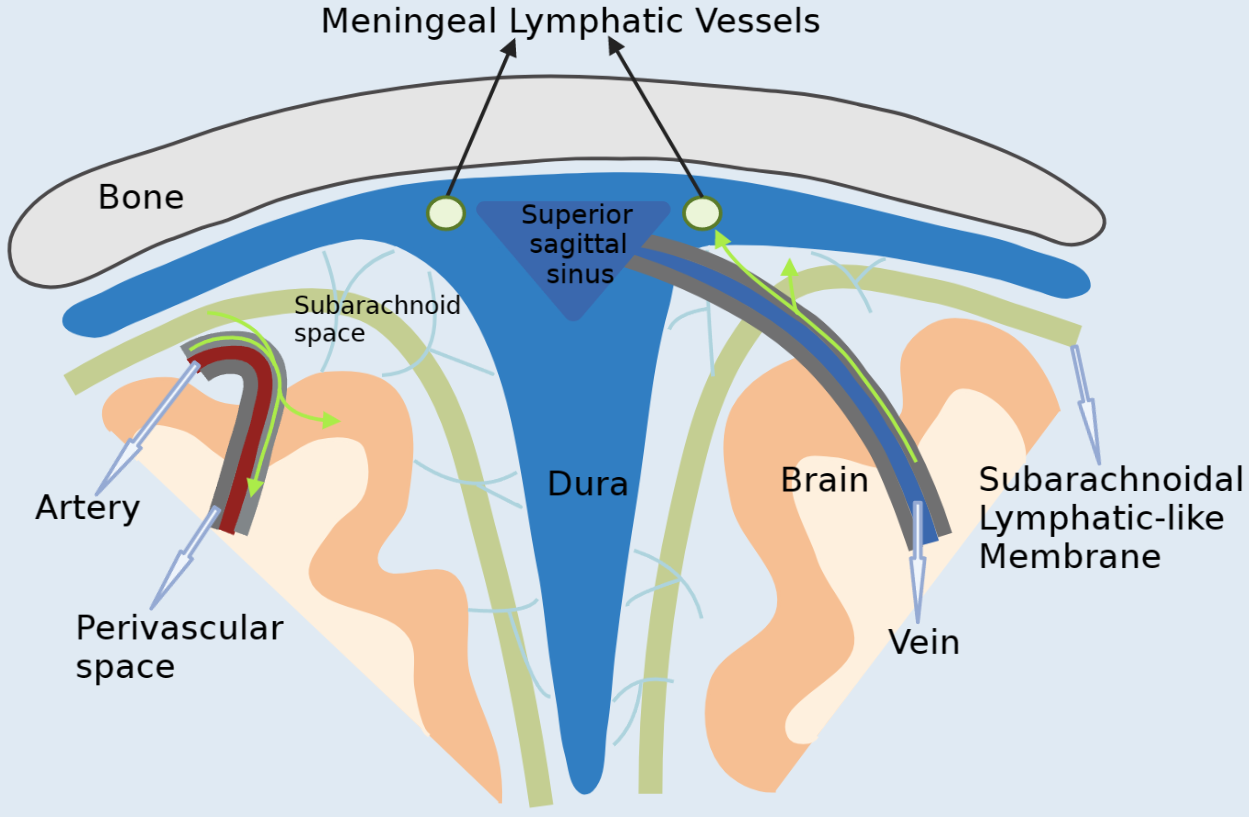
**Basic information about meningeal lymphatic vessels**. Cerebrospinal fluid from the subarachnoid space enters the brain along the periarterial space, after which it leaves the periarterial space to exchange with the ISF in the brain parenchyma. It then exits along the perivenous space and returns to the subarachnoid space. A portion of this returns to the cerebrospinal fluid and a portion enters meningeal lymphatic vessels.

The specific functions of the meningeal lymphatic vessels differ depending on the location of their distribution. To date, several location-specific meningeal lymphatic systems have been studied in a targeted manner, such as basal MLVs, and dorsal MLVs [17, 27, 28]. The dorsal MLVs travel along the superior sagittal sinus (SSS) and the transverse sinus (TS). Their lumen diameter is small, most of them do not form branches, and they do not have valvular structures in their lumen. Their endothelial cells are interconnected in a discontinuous closed, loosely button-like fashion. In contrast, basal MLVs travel along the phosphoric sinus (PSS) and sigmoid sinus (SS). Their lumen diameter is large, with abundant capillary branches and rounded ends. Their endothelial cells have a typical oak leaf shape, and valves are present in the lumen. Moreover, in terms of their distribution, basal MLVs are closer to the subarachnoid space than dorsal MLVs. The anatomical differences between the basal MLVs and the dorsal MLVs determine that the former is more suitable for the drainage of large molecules in the cerebrospinal fluid. And the results of the study also proved that the basal MLVs are the main channels for cerebrospinal fluid drainage [27]. The study by Xue-ting Hu and her team showed that intracranial tumors were able to induce extensive remodeling of dorsal MLVs and were able to induce mild remodeling of basal MLVs in advanced stages [28]. This is a very valuable finding that the physiological functions of meningeal lymphatic vessels distributed in different sites may be somewhat different and require some differentiation in the study. For example, in studies addressing changes in meningeal lymphatic vessels with age, it was found that the function of dorsal MLVs consistently showed a trend of decay with increasing age in mice. In contrast, the basal MLVs changed less and even showed a compensatory enhancement in older mice. This fully demonstrates the functional differences between the two due to the different anatomical structures and distribution sites [27]. Therefore, targeted modulation of specific sites of meningeal lymphatics under pathological conditions may help us to greatly improve therapeutic efficiency and achieve better therapeutic benefits. It is also important to note that although the above studies mainly demonstrated the important role of basal MLVs in intracranial drainage, this does not mean that dorsal MLVs are less important. The two may simply have different functional emphases. At present, our understanding of the fine structure of meningeal lymphatics is still very limited, and a lot of relevant studies are still needed to explore it. The structural and compositional differences between different sites of meningeal lymphatics may inspire us to explore more potential targets, such as a specific receptor on the endothelial cells of a particular site of meningeal lymphatics. Another important issue is that the structure of the meningeal lymphatic vessels has been shown to change to some extent with age. And this alteration usually has a negative effect, which makes the risk of developing many types of diseases, such as neurodegenerative diseases [12, 29-31]. Therefore, after clarifying the meticulous changes in the structure of the meningeal lymphatic vessels with age, attempts to reverse this change during treatment might provide great therapeutic benefit.

**Figure 2.**
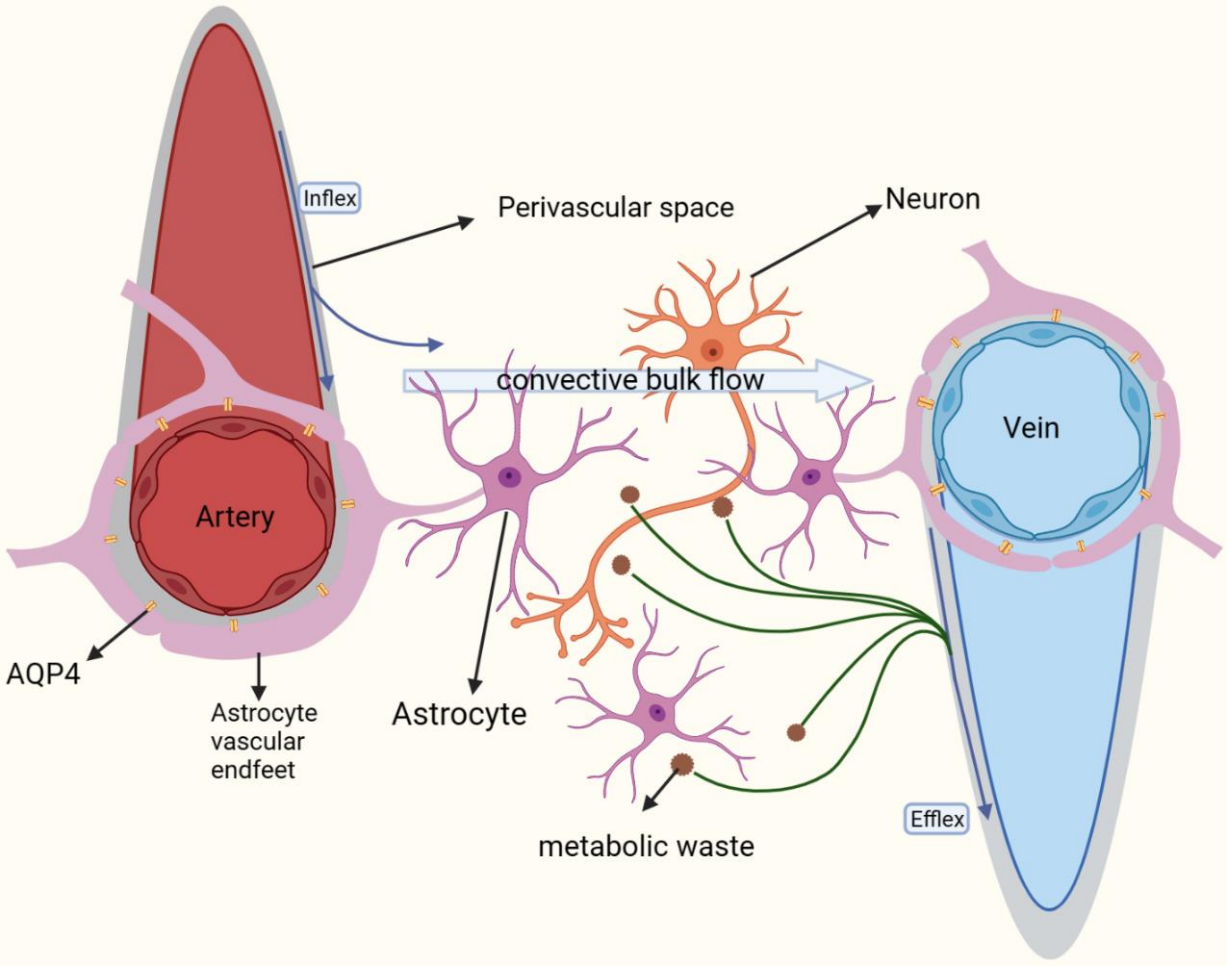
**Basic information about glymphatic system**. The mass flow of interstitial solutes through the brain parenchyma through pressure gradients. In this way, metabolic waste products such as toxic proteins in the brain parenchyma can be effectively removed. This can effectively maintain brain homeostasis.

### 2.2 Perivascular spaces

Perivascular spaces are a particular class of structures that exist around the blood vessels of the brain, which are filled with interstitial fluid [32, 33]. The outer boundary of the perivascular spaces consists of a glial boundary membrane. It is a compact structure formed by astrocyte foot processes and the parenchymal basement membrane that covers them. The inner boundary of the perivascular spaces, on the other hand, consists of the basement membrane of the vascular endothelium. The inner boundary and the outer boundary together extend with the vascular tree all the way to the capillary network. Finally, the two fuse to form the blind end [34-37]. Depending on their location, perivascular spaces can be divided into three types: basal ganglia type, hemispheric type and midbrain type [38]. However, the specific structural and functional differences between these three types remain to be investigated. As an important component of the glymphatic system, the perivascular spaces may be deeply involved in fluid drainage and metabolic waste removal in the brain [39-41]. Rennels ML et al. showed that cerebrospinal fluid (CSF) first enters the brain parenchyma from the subarachnoid space via the periarterial spaces. It then exchanges fluid with interstitial fluid (ISF). Finally, the fluid flow returns through the perivenous spaces to the subarachnoid space [42]. In this process, wastes removal from the perivascular spaces rely primarily on microglia, pericytes and perivascular phagocytes [43-45]. It is important to note that there are also views that some perivascular spaces do not directly communicate with the subarachnoid space [33, 42, 46-48]. The specific processes and mechanisms by which the cerebrospinal fluid interacts with the interstitial fluid in the perivascular spaces of different types of vessels remain to be investigated. In addition, a drainage pathway called "intramural periarterial drainage" has also been identified. In this pathway, the basement membrane of the cerebral arteries or capillaries appears to play a similar role to that of the cerebral lymphatic system. The process is also highly dependent on sustained cardiac output [49, 50]. However, whether this particular pathway is also present in the perivascular spaces remains to be investigated further. Numerous studies have found that enlargement of the perivascular spaces may be present in several types of central nervous system diseases, such as Alzheimer's disease, neurofibromas and familial brain malformations. This may be related to factors such as damage to the cerebral microvasculature caused by the accumulation of metabolic waste [51-54]. In addition, Park CH's study revealed an age-dependent dilatation of the perivascular spaces, with a particularly marked dilatation of the basal ganglia type [55]. This demonstrates that ageing may be similarly strongly associated with the enlargement of the perivascular spaces [56-58]. Iliff JJ showed that ageing also causes a slowing of fluid flow within the perivascular spaces and hardening of the arterial wall [40]. However, there is still a lack of follow-up studies on the enlargement of the perivascular spaces in large samples over a long period of time. This remains to be further explored.

### 2.3 Astrocytic endfeet

The astrocyte endfeet are special subcellular structures emanating from astrocytes, which are distributed with a large amount of aquaporin-4 [59]. They play an important role in regulating brain homeostasis, maintaining the integrity of the blood-brain barrier and removing waste solutes [60, 61]. Numerous studies have shown that astrocytes are an important component of the cerebral lymphatic system [62, 63]. Astrocyte endfeet are involved in forming the outer wall of the perivascular spaces, which in turn form the glymphatic system [64].

Aquaporin-4 (AQP-4) is a tetrameric structure with a molecular weight of 30 kDa and a pore size of approximately 0.5 nm. AQP-4 is predominantly found in the endfeet of astrocytes around brain vessels and is usually polarized [65, 66]. Galina Yankova et al. showed that the endfeet of astrocytes have a wide coverage and that fluid and solutes must pass through the gap between the endfeet of AQP-4 or astrocytes in order to penetrate outside the perivascular spaces. Interestingly, the cellular gap between the end feet is often less than 20 nm, which implies that the fluid flow transfer pathway through AQP-4 may play a major role [67]. In fact, numerous studies have shown that AQP-4 on the astrocyte endfeet promotes fluid flow between the cerebrospinal fluid and interstitial fluid [3, 21, 65, 68, 69]. Yifan Wang et al. found that a decrease in AQP-4 may likewise disrupt the normal structure and function of astrocytes, leading to an impaired establishment of a reactive astrocytic network [21, 61, 65, 68, 70]. Nadia Aalling Jessen and Kazuhisa Ishida et al. demonstrated that the drainage of cerebrospinal fluid was severely impaired in AQP-4 knockout mice compared to wild group mice. This resulted in significantly increased levels of β-amyloid and tau proteins in the cerebrospinal fluid, which further exacerbated the associated neurodegenerative pathologies [21, 71]. However, the specific distribution of AQP-4 on astrocytes and the potential mechanisms by which it mediates the transport of fluids and solutes remain to be investigated [65, 69]. In addition, AQP-4 has an important functional feature of circadian rhythm [72]. Fluid flow and clearance of metabolic waste in the cerebral lymphatic system are both significantly enhanced and peak during deep sleep [73]. Lauren M. Hablitz et al. showed that the distribution of cerebrospinal fluid is controlled by circadian rhythms and regulated by AQP-4[74]. Therefore, studying the underlying mechanisms of the circadian rhythm of AQP-4 may help us to better explore the functional characteristics of AQP-4.

## 3. Functional characteristics of the cerebral lymphatic system

### 3.1 Role in regulating cerebrospinal fluid circulation

Cerebrospinal fluid is produced by the choroid plexus, brain interstitium, and meninges and is stored in the ventricular and subarachnoid spaces of the brain. It has a variety of functions including the transport of nutrients and metabolic waste, immunity and the regulation of intracranial pressure. The efficient circulation of cerebrospinal fluid is important for the maintenance of environmental homeostasis in the central nervous system [75, 76]. However, with further understanding of the cerebral lymphatic system, we are aware that the old hypothesis of cerebrospinal fluid circulation may be somewhat problematic. The presence of the cerebral lymphatic system has to some extent broadened the range of fluid and solute flow in the cerebrospinal fluid and enriched the pathways of cerebrospinal fluid circulation. This is mainly achieved by the glymphatic system [11, 12, 27, 77]. The current view is that substances in the CSF are able to enter the perivascular spaces of the arteries and enter the brain parenchyma via AQP-4 in the peduncle of astrocytes. The Bulat-Klarica-Oreˇskovi’c hypothesis proposes that the bidirectional exchange of solutes and fluids between cerebrospinal fluid and interstitial fluid via AQP-4 and the flow of fluid streams is continuous. Osmotic and hydrostatic pressures are the major drivers of this process [77-79]. Subsequently, the material follows the fluid flow in the interstitial fluid to the relatively hypertonic perivascular spaces. Thereafter, the fluid flow in the perivascular spaces can drain along the nerve sheaths of the cranial and spinal nerves, the arachnoid granules and the meningeal lymphatic vessels [3, 7, 80-86]. Many studies have shown that the cerebrospinal fluid outflow from the CNS through the dorsal and basal dural lymphatics in the cerebral lymphatic system accounts for 30-50% of the total cerebrospinal fluid drainage [8, 12, 87]. This demonstrates the importance of the cerebral lymphatic system in the process of cerebrospinal fluid circulation. Considering the immune function of the cerebral lymphatic system, by enhancing the function of the cerebral lymphatic system, we may be able to find more effective and complete solutions for the removal of harmful waste products from the central nervous system.

### 3.2 Macromolecule and immune cell transport

Recent studies have found that the cerebral lymphatic system plays an important role in the transport of macromolecular substances [88]. Conventional wisdom holds that the blood-brain barrier (BBB) to some extent impedes the entry of macromolecular substances into the brain [89]. However, the cerebral lymphatic system well bypasses the obstruction of the blood-brain barrier and provides a new pathway for the transport of macromolecular substances. On the one hand, the cerebral lymphatic system is able to transport and supply nutrients to the tissues and cells of the central nervous system [88], such as glucose [90], folic acid [91], lactate [92], and vitamins [91]. Notably, this pathway may be the only route to the CNS for some nutrients that cannot transit through the blood-brain barrier, such as glutamate [93]. On the other hand, the cerebral lymphatic system also takes on the important function of removing metabolic waste products produced by the CNS [94], such as soluble β-amyloid [3], tau protein [95] and α-synuclein [7]. However, further research is needed on the types of substances that are transported through the cerebral lymphatic system and the specific mechanisms of the transport process [96, 97]. In addition, the cerebral lymphatic system can also facilitate the movement of viruses into and out of the central nervous system. And this has a certain double-edged effect. On the one hand, peripheral viral infections may invade the CNS through the cerebral lymphatic system. This may be achieved through the blood-brain barrier, the nasal lymphatics or the meningeal lymphatic vessels [98]. On the other hand, meningeal lymphatic vessels may divert neurotropic viruses from the CNS to the deep cervical lymph nodes, thereby reducing CNS damage [99]. However, it is important to note that this process may also induce a peripheral immune response, which in turn may lead to inflammation and injury in the peripheral system [100]. Therefore, it is important to explore how to find a balance between these two effects.

Furthermore, the cerebral lymphatic system also has the function of transporting immune cells [11]. The cerebral lymphatic system is responsible for the transport of many types of immune cells, such as macrophages, dendritic cells, T cells, B cells, monocytes, neutrophils, etc. [11, 101-105]. Among these, the immune cells in the dura mater are more heterogeneous compared to those in other sites. This shows a certain peculiarity. After entering the cerebrospinal fluid, cytokines produced by T cells distributed in the dura mater may enter the brain parenchyma directly through the perivascular spaces to activate neurons. In addition, they may also indirectly regulate neuronal activity by inducing the secretion of other cytokines by glial cells [101, 106-111]. However, given that the dura mater is not in direct contact with the brain parenchyma, the exact pathways and mechanisms by which cytokines cross the arachnoid and enter the cerebrospinal fluid are still unclear. At the same time, the immune system of the central nervous system is able to interact with the peripheral immune system through the cerebral lymphatic system [112]. It is important to note that the immune response within the CNS differs somewhat from that in other tissues [113-116], but the underlying mechanisms remain to be explored. In addition, a recent study by Maiken Nedergaard's team identified a new structure in the brain, the subarachnoid lymphoid membrane (SLYM), which is a membrane structure composed of a layer of Prox1 protein-containing cells interwoven with collagen fibres. It is capable of dividing the subarachnoid space into an outer superficial chamber and an internal deep chamber [6]. Under physiological conditions, the SLYM not only mediates the controlled flow of cerebrospinal fluid and the exchange of substances, but it also contains immune cells that play an immunodetective role. When the homeostasis of the brain is disrupted, a large number of immune cells cross the SLMY and reach the brain parenchyma, causing neuroinflammation [117, 118]. Further studies of the SLMY may provide a better understanding of the flow of fluid in the brain and the mechanisms underlying the movement of immune cells in the brain.

### 3.3 Substance exchange between the central nervous system and peripheral system

Traditionally, the central nervous system is thought to be able to exchange substances with the periphery via the blood and cerebrospinal fluid, and this process is regulated primarily by the blood-brain and blood-cerebrospinal fluid barriers [119]. Studies have shown that 40% of Aβ in the brain can be transported to the peripheral system for clearance [3, 41, 43]. At the same time, peripheral injection of brain extracts containing large amounts of Aβ also induced excessive accumulation of Aβ in the brains of humans and mice [120-123]. This suggests that there may be a close exchange of substances between the central nervous system and the peripheral system. However, as we have learned more about the cerebral lymphatic system, we have discovered that it may also play an important role in the exchange of substances between the central nervous system and the peripheral system. Shinji Naganawa et al. found that when gadolinium-based contrast agents (GBCAs) were injected intravenously, GBCAs could bypass the blood-brain barrier and be transported through the cerebral lymphatic system into the brain parenchyma [124, 125]. However, the exact flow pathways and mechanisms of this process remain unclear. Notably, there may be a special link in the process of material interaction between the CNS and the peripheral system, i.e., a possible bidirectional traffic between the meningeal lymphatics and the deep cervical lymph nodes. This differs significantly from the familiar unidirectional transport of peripheral lymphatic vessels. The study showed that after injection of 99mTc-DX into the deep cervical lymph nodes, Fan Chen et al. detected radioactivity in the skull base dura, cerebrospinal fluid and brain tissue. Moreover, radioactivity could be detected in the skull base dura before brain tissue [126]. This may provide indirect evidence for a bidirectional interaction between the peripheral and cerebral lymphatic systems. Another possible route for the reentry of drainage fluid from the cervical lymph nodes into the brain may now be via blood circulation and finally across the blood-brain barrier. However, the exact pathway of how cervical lymph node drainage reenters the brain is currently unknown.

## 4. The critical role of the cerebral lymphatic system as a bridge between the peripheral system and the central nervous system

Numerous studies have shown that the cerebral lymphatic system is extensively and closely linked to the peripheral system. Some peripheral organs, such as the liver, gastrointestinal tract, and kidney, may be involved in the potential peripheral clearance pathways of toxic proteins from the central nervous system [127-131]. For example, as a classic neurodegenerative disease, Alzheimer's disease is a classic case of communication between the cerebral lymphatic system and the peripheral system [132]. The abnormal accumulation of toxic proteins such as amyloid-β and tau in the brain due to the imbalance between production and clearance is considered as the main causative factor [133-139]. And according to experimental results, 19% of pathological tau proteins [140] and 40% of Aβ in the brain are transported to the periphery for clearance [127]. Therefore, some alterations in the peripheral system may lead to abnormalities in the central nervous system through the cerebral lymphatic system as a mediator.

### 4.1 Communication and regulation between the central nervous system and the gastrointestinal tract via the cerebral lymphatic system

The main function of the gastrointestinal tract is the secretion of digestive enzymes and the absorption of ingested nutrients. This is mainly regulated by the central nervous system (CNS), the enteric nervous system (ENS) and enteroendocrine cells [141, 142]. Currently, several CNS disorders have been shown to be associated with the gastrointestinal tract and may even originate in the gastrointestinal system, such as Alzheimer's disease, Parkinson's disease, and ischemia/reperfusion injury [143-148]. Recent insights suggest that bidirectional effects exist between components of the microbial-brain-gut axis and are achieved through multiple neural, hormonal, metabolic, and immune mechanisms [149-153]. And in this process, the cerebral lymphatic system may be the key intermediate link between the brain and the gastrointestinal tract to communicate with each other. On the one hand, the gastrointestinal tract may have an impact on the function of the brain lymphatic system. It was found that in a mouse model of colitis induced with dextran sodium sulfate (DSS), enhanced NLRP3 inflammatory vesicle activity induced neuroinflammation and impaired astrocyte function. This leads to reduced clearance of the brain lymphatic system, which causes cognitive dysfunction in mice [14, 154-156]. Also, dysbiosis of gut microbes induces disturbances in cerebrospinal fluid flow, which triggers a decrease in the function of the cerebral lymphatic system [157]. In a mouse model of Porphyromonas gingivalis (Pg) gavage, we also observed a reduction in the rate of CSF-ISF exchange and ISF flow in the brain. This is thought to be related to the dysfunction of the cerebral lymphatic system [15].

**Figure 3.**
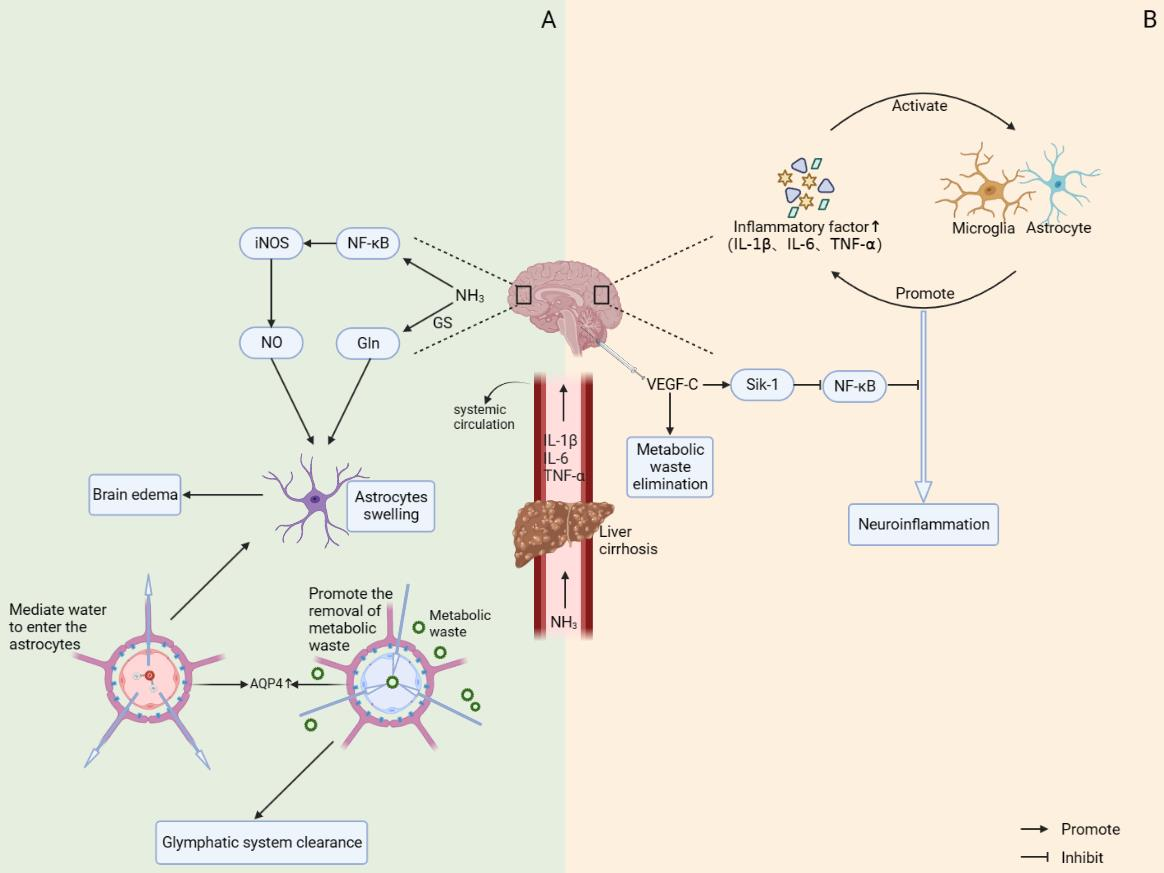
**Possible mechanisms of co-occurring neuroinflammatory response and brain edema in patients with hepatic encephalopathy and potential therapeutic targets for hepatic encephalopathy**. Decreased detoxification of the liver in patients with cirrhosis leads to the accumulation of NH_3_ and inflammatory factors (IL-1β, IL-6, TNF-α) in their bodies. These substances can accumulate in the brain tissue with blood circulation and subsequently induce hepatic encephalopathy. (**A**) On the one hand, excess NH_3_ in the brain is converted to Gln by GS, causing a hyperosmotic environment in astrocytes. On the other hand, it can increase the activity of iNOS by activating NF-kB pathway, which makes the level of NO elevated. This can lead to astrocyte swelling and eventually brain edema. Meanwhile, targeted regulation of AQP-4 expression levels has a dual role. Increased AQP-4 expression not only promotes the drainage of metabolic waste from glymphatic system, but also mediates the entry of water into the astrocytes thus causing brain edema. (**B**) Increased inflammatory factors can activate microglia and astrocytes. Activated microglia and astrocytes in turn further promote the release of inflammatory factors. Positive feedback is formed between the two. Exogenous injection of VEGF-C may inhibit the NF-kB signaling pathway by upregulating the expression of Sik-1, which in turn reduces the inflammatory response. At the same time, VEGF-C may also promote the clearance of metabolic wastes such as NH_3_ by inducing the proliferation of meningeal lymphatic vessels.

In addition, metabolites of microorganisms may also have an effect on the cerebral lymphatic system. Some bacterial metabolites have been found to positively affect astrocytes, such as sodium butyrate that exacerbates MPTP-induced astrocyte activation in a mouse model of Parkinson's disease, thereby exacerbating neuro-inflammation and colitis [158]. However, some other bacterial metabolites have neuroprotective effects. For example, mucilaginous extracts of Archangium sp. UTMC 4070 and Cystobacter sp. UTMC 4073 strains could exert anti-oxidative stress effects to protect astrocytes. On the other hand, the cerebral lymphatic system may also provide a pathway for the transfer of bacteria and bacterial products, inflammatory factors and immune cells from the gastrointestinal tract to the center. Bostancıklıoğlu M suggested that the virus may enter the central nervous system via the lymphatic system based on the correlation between neurological and digestive symptoms in patients with novel coronavirus pneumonia [159, 160]. Similarly, Porphyromonas gingivalis may also cause brain inflammation through the cerebral lymphatic system [161, 162]. In dysbiosis of the gastrointestinal flora, the microbiota and its products stimulate the production of cytokines by gastrointestinal epithelial cells and macrophages. And the cerebral lymphatic system may provide a pathway for these cytokines to reach the brain [163]. On the other hand, during gastrointestinal flora dysbiosis, microbial-associated molecular patterns (MAMP) and pathogen-associated molecular patterns (PAMP) of the gut microbiota can be recognized by pattern recognition receptors on immune cells in the central nervous system. This may induce the release of inflammatory cytokines and affect the activity of astrocytes, which in turn may have an impact on the cerebral lymphatic system. Toll-like receptors recognize microbial nucleic acids, lipopolysaccharides, pepti-doglycans, and other components to signal to downstream inflammatory pathways, including NF-κB, MAPK, and/or interferon-regulatory factor signaling pathways [164]. In contrast, activation of NF-κB-related pathways mediated by NLRP3 inflammatory vesicles may reduce the polarity of AQP-4, which in turn leads to impaired normal function of the brain lymphatic system [14, 65, 165-168]. In addition, TREM receptors are potential mediators of the gastrointestinal tract affecting the brain through the cerebral lymphatic system [169]. TREM-2 is able to exert anti-inflammatory effects by negatively regulating Toll-like receptor 4 in astrocytes [170, 171].

Endocrine hormones, especially those involved in the hypothalamic-pituitary-adrenal axis, are important components of the brain-gut axis. They are able to influence the cerebral lymphatic system by participating in the regulation of astrocyte morphology and function, which may in turn [82, 151, 172-174]. Previously, the gut microbiota has been shown to influence the expression of genes associated with the HPA axis [175, 176]. Maria Meyer's study showed that adrenal glucocorticoids may trigger neuroinflammation by activating Toll-like receptor 4 on astrocytes, which subsequently triggers neuroinflammation. Adrenocorticotropin-releasing hormone can increase AQP-4 expression via the cAMP / PKA signaling pathway, which in turn causes astrocyte swelling [177, 178]. In addition, Yu-Xia Lou et al. found that adrenal glucocorticoids were able to impair the normal function of the gap junction between astrocytes [179]. This may likewise affect the drainage function of the cerebral lymphatic system.

When the ecology of the gastrointestinal tract is dysregulated, neural signals generated in the gastrointestinal tract are transmitted to the brain via afferent nerves. And the process produces certain neurotransmitters that may be involved in the regulation of astrocytes [172, 180]. For example, serotonin can be taken up by astrocytes and regulate their functions [181, 182]. And 90-95% of serotonin is produced in the gastrointestinal tract [183]. Some selective serotonin reuptake inhibitors (SSRI) have been found to inhibit the inflammatory response of astrocytes by inhibiting the NF-κB pathway, which in turn may have an effect on the cerebral lymphatic system [183-185].

### 4.2 Communication and regulation between the central nervous system and the liver via the cerebral lymphatic system

As a detoxification organ in the body, the liver plays an important role in the removal of toxins from the body [186, 187]. It plays an irreplaceable role in the metabolism and detoxification of NH_3_. When the liver is dysfunctional, this function can be significantly reduced, which can cause the accumulation of toxic products such as NH_3_ and cause serious damage to vital organs of the body, such as brain [188, 189]. Therefore, accelerating the clearance of toxic metabolites may be important for the treatment of this type of disease. As a new concept proposed in recent years, the cerebral lymphatic system has a role in drainage and removal of metabolic waste, immune monitoring, etc. This is highly similar to the functions of the liver. Numerous studies have also shown a solid link between the two.

#### 4.2.1 The relationship between the cerebral lymphatic system and hepatic encephalopathy

Hepatic encephalopathy (HE) is a serious complication that occurs in the end-stage of various liver diseases such as cirrhosis. It is mainly caused by the weakened detoxification function of the liver and elevated blood ammonia due to portal-systemic shunting [190]. Besides, neuroinflammation is also an important causative factor of HE [191]. It has been shown that patients with cirrhosis are often accompanied by systemic inflammatory response syndrome [192]. These inflammatory factors activate microglia and astrocytes and prompt them to further release pro-inflammatory factors [193]. Positive feedback is formed between the two, which in turn exacerbates the neuroinflammatory response in the brain. Therefore, by promoting the excretion of NH_3_ and other metabolic wastes in the brain of patients with hepatic encephalopathy, it has some therapeutic significance for the neuroinflammatory response in the brain. The functional characteristics of the cerebral lymphatic system to remove and drain metabolic wastes make it a potential target for the treatment of hepatic encephalopathy.

**Figure 4.**
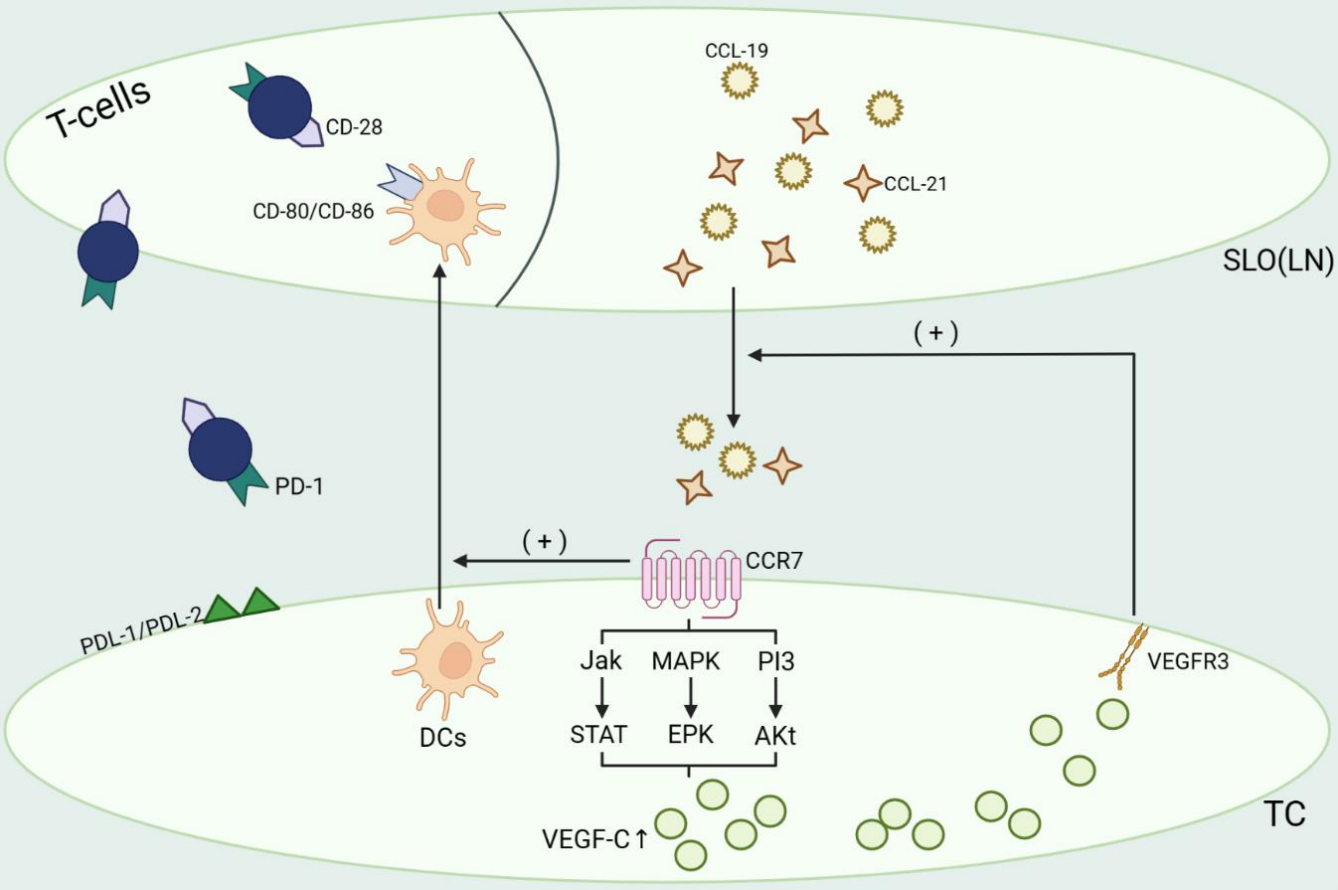
**The binding of CCL19/CCL21 to CCR7 activated signaling pathways in tumor cells, including PI3/Akt, MAPK/ERK and Jak/STAT, which enhanced the expression of VEGF-C and thus promoted lymphangiogenesis**. Meanwhile, activation of the VEGF-C/VEGFR-3 signaling pathway can promote the release of CCL19 and CCL21 in lymph nodes. Furthermore, activation of the CCR7 chemokine axis can promote dendritic cell entry into the T cell compartment of lymph nodes, which enables specific recognition of CD80/CD86 on dendritic cells and CD28 on T cells. This activates T cells and promotes T cell infiltration into the tumour.

Aspelund et al. demonstrated that hypoplasia of the meningeal lymphatics leads to a significant reduction in the clearance of meningeal lymphatics in mice [7]. Shao-Jung Hsu and his team effectively promoted the drainage of metabolic waste from the brain by promoting the generation of meningeal lymphatics in a mouse model suffering from cirrhosis with hepatic encephalopathy. This helped alleviate the microglia activation and neuroinflammatory responses, thus ameliorating the symptoms of hepatic encephalopathy to some extent [13]. In addition to this, Pasquale Gallina et al. proposed a hydrodynamic hypothesis regarding the pathogenesis of glymphatic system damage in patients with hepatic encephalopathy [194]. Another study showed that defects in the glymphatic system led to reduced clearance of harmful substances in the brain of patients with cirrhosis. And its mechanism of action may be related to the decreased expression of AQP-4 [195]. Therefore, upregulation of AQP-4 may be able to promote the removal of toxic waste by the glymphatic system. This shows that the cerebral lymphatic system has great potential to become a treatment for hepatic encephalopathy.

#### 4.2.2 Dual role of cerebral lymphatic system in brain metastasis from hepatocellular carcinoma

Brain metastasis from hepatocellular carcinoma is an important cause of death in patients with hepatocellular carcinoma. The current treatment options for it are relatively limited and the prognosis is generally poor [196, 197]. It is currently believed that most brain metastasis from tumors occur through hematogenous metastasis [198]. However, with the introduction of the concept of cerebral lymphatic system, we may be able to further improve the body of knowledge on brain metastasis from liver tumor. Numerous studies have shown that the CCL21/CCR7 axis plays an important role in the lymphatic metastasis of tumors [199, 200]. On the one hand, CCL21 interacts with CCR7 to control the transfer of immune cells such as DCs and T cells to lymphatic vessels and lymph nodes, which in turn enhances the anti-tumor immune response [201]. This is beneficial for tumor treatment. Xueting Hu and his team demonstrated that the combination of anti-PD-1/CTLA4 therapy showed better therapeutic effects after overexpression of VEGF-C in the intracranial tumor mouse model [28]. However, this effect was reversed after blocking the CCL21/CCR7 axis. This suggests that the effect of VEGF-C-enhanced antitumor immunotherapy is somewhat dependent on the CCL21/CCR7 axis. On the other hand, certain CCR7-expressing tumor cells can also use this to undergo lymphatic metastasis, leading to tumor spread and metastasis [202].

**Figure 5.**
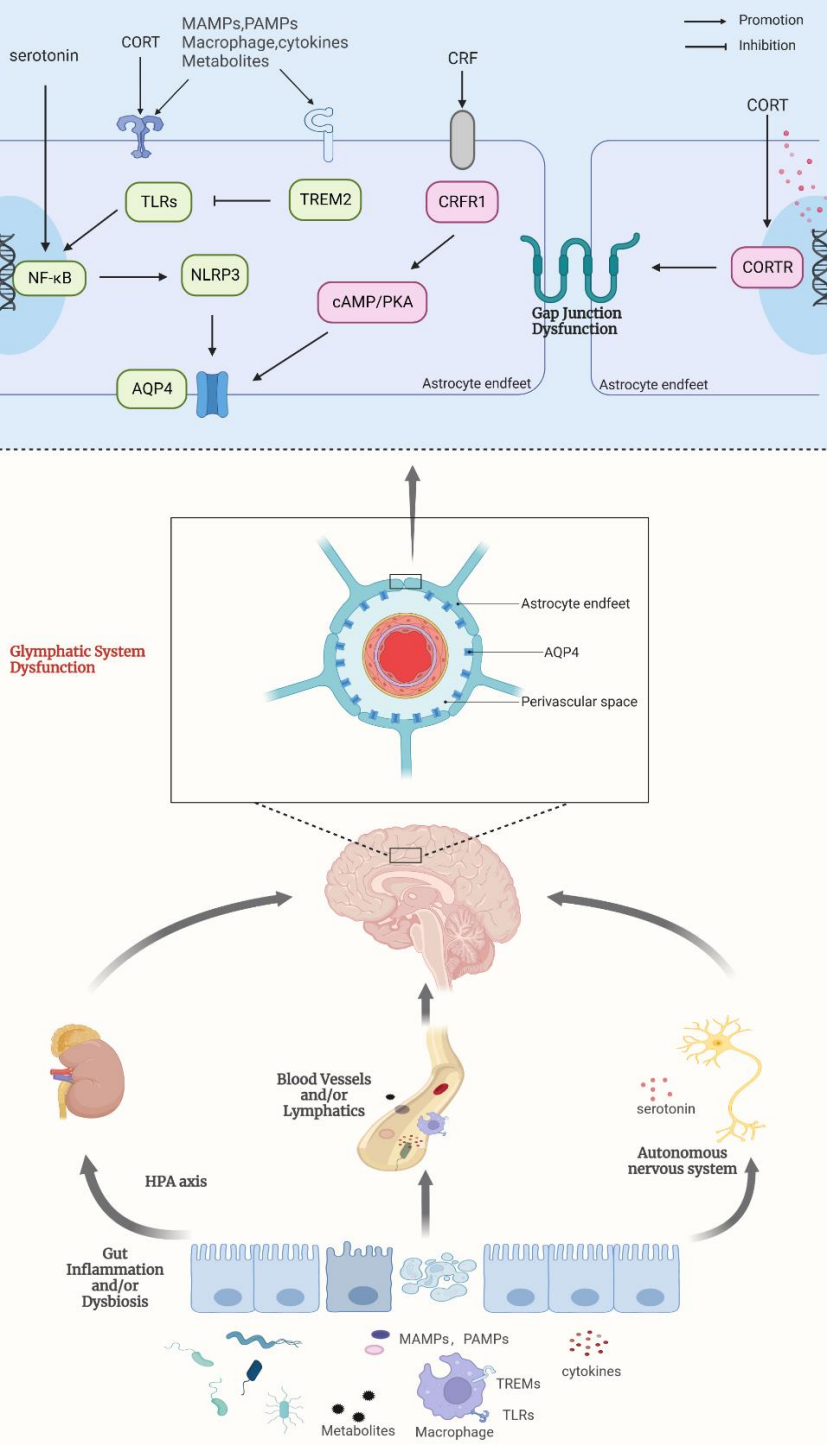
**Communication between the gastrointestinal tract and cerebral lymphatic system is mediated directly and indirectly by immune, neural and endocrine pathways**. (**A**) Changes in the gut microbiota occur when inflammatory damage occurs in the gut. Pathogenic microorganisms and their products, inflammatory factors, pathogen-associated molecular patterns (MAMPs, PAMPs), TLRs, and TREMs-positive activated macrophages may reach the brain through blood circulation and lymphatic circulation, which in turn affect the function of cerebral lymphatic system. (**B**) Some neurotransmitters (e.g., serotonin) also act on astrocytes and lead to activation of related pathways, which in turn affects the function of cerebral lymphatic system. (**C**) The gut microbiota can regulate hormones related to the hypothalamic-pituitary-adrenal axis, which in turn affects the function of cerebral lymphatic system.

Ding et al. found an association between high CCR7 expression and lymph node metastasis and poor prognosis in esophageal squamous cell carcinoma [203]. VEGF-C overexpression induces the formation of neo-lymphatic vessel [204], which undoubtedly facilitates the metastasis of hepatocellular carcinoma to the brain. However, a study showed that the incidence of brain metastasis in lung cancer patients showed a significant positive correlation with the high expression of VEGF-C [205]. So does it have similar results in patients with brain metastasis from hepatocellular carcinoma? However, there is still a lack of validated studies proving the relationship between brain metastasis of hepatocellular carcinoma and high expression of VEGF-C. Therefore, our team hypothesized that targeted regulation of VEGF-C and CCR7 may become a novel therapeutic option for the prevention and treatment of brain metastasis via lymphatic route in patients with hepatocellular carcinoma in the future. And it becomes crucial to master a balance between VEGF-C in enhancing anti-tumor immune response and promoting tumor metastasis.

### 4.3 Communication and regulation between the central nervous system and the kidney via the cerebral lymphatic system

The kidney is able to eliminate metabolic wastes from the body by producing urine, which in turn maintains the homeostasis of the body's internal environment [206]. In recent years, as the kidney-brain axis continues to receive widespread attention, the anatomical and patho-physiological connections between the kidney and the brain have been further revealed [207-213]. Interestingly, the cerebral lymphatic system is able to facilitate the removal of metabolic waste by deeply engaging in fluid circulation in the brain [13]. This is highly similar to the function of the kidney. Currently, numerous studies have shown that the cerebral lymphatic system is an important "bridge" between kidney and brain.

**Figure 6.**
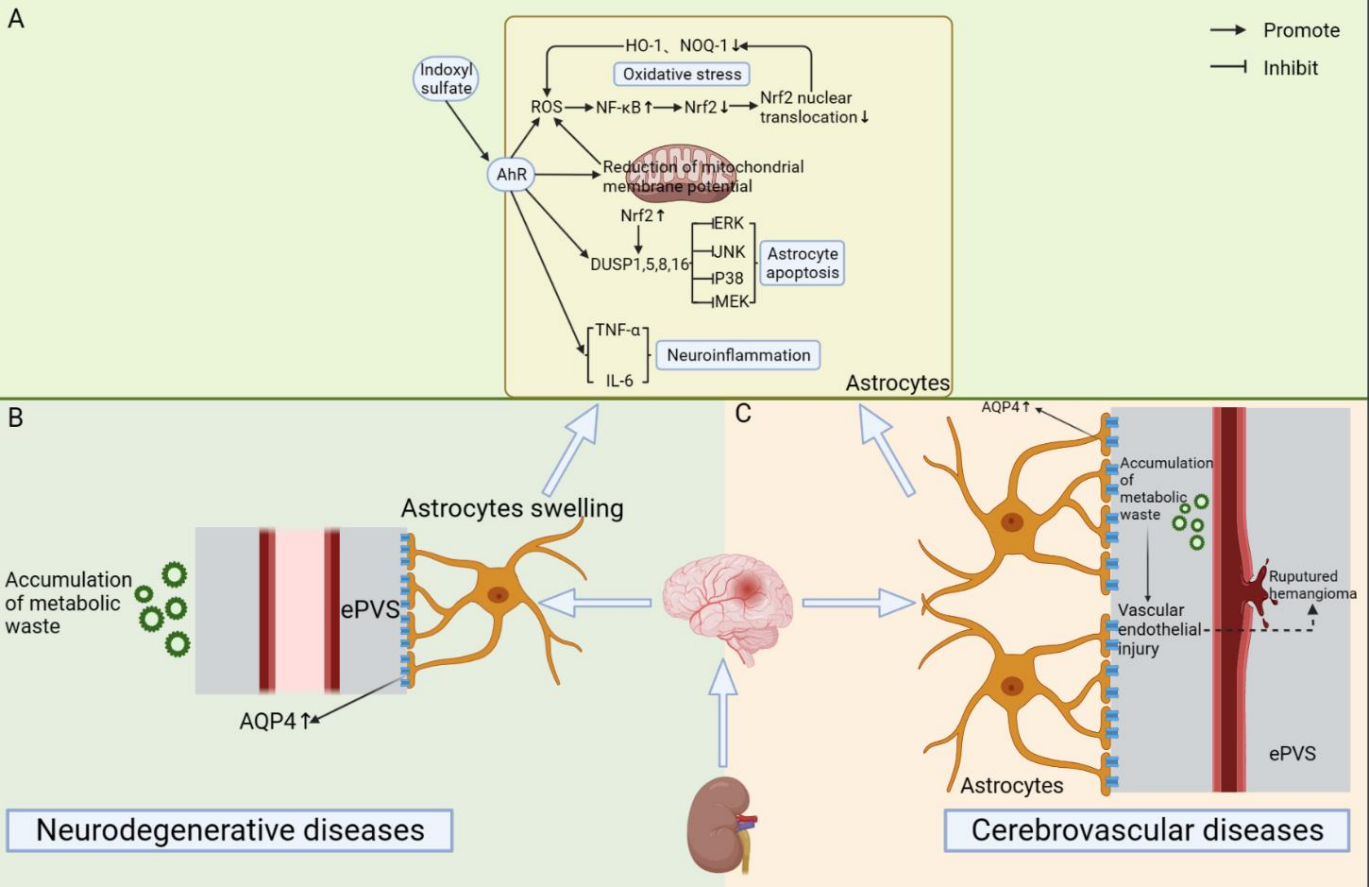
**Mechanism of action of cerebral lymphatic system in brain diseases due to impaired renal function**. (**A**) Indoxyl sulfate (IS) accumulates during impaired renal function and activates its receptor AhR (Aryl hydrocarbon Receptor), which in turn activates downstream oxidative stress, inflammation and apoptotic pathways. This ultimately leads to the damage of astrocytes. This is a common mechanism for the development of kidney-induced neurodegenerative and cerebrovascular diseases. (**B**) The accumulation of metabolic wastes in the brain is able to increase the expression of AQP-4 and widen the perivascular spaces. This induces the development of neurodegenerative diseases. (**C**) The accumulation of metabolic wastes in the brain can cause endothelial cell damage, increased AQP-4 expression, and the enlargement of perivascular spaces. This can induce the development of cerebrovascular diseases such as intracranial aneurysms.

#### 4.3.1 The role of the cerebral lymphatic system as a bridge between renal disease and cerebrovascular disease

Numerous studies have shown that there are certain similarities in the anatomy and blood supply of kidney and the brain. As low resistance end organs that are also subject to high flow blood shock, both have a greater potential for microvascular injury [16, 207]. The cerebral lymphatic system plays an important role in the interplay of renal and cerebrovascular diseases. On the one hand, renal disease may promote the development of cerebrovascular disease. In the case of chronic kidney disease (CKD), its promotion of cerebrovascular disease is mainly attributed to the damaging effect on cerebrovascular endothelial cells caused by the accumulation of metabolic waste products (e.g., indolol sulfate) in the body [214, 215]. A study by Ashley A Penton and her team suggested that CKD may be a potential trigger for the enlargement of the perivascular gap in the brain in stroke patients. And the enlargement of the perivascular spaces may be highly associated with the dysfunction of the glymphatic system [216, 217]. Therefore, the cerebral lymphatic system may play a role in the induction of cerebrovascular disease by renal disease. On the other hand, cerebrovascular disease may promote the development of renal disease. Some studies have found that the incidence of CKD is much higher in stroke patients than in the general population [218]. However, the underlying mechanism by which cerebrovascular disease induces the development of renal disease is unclear. We speculate that this may be caused by the impaired function of the central nervous system leading to the normal physiological function of the kidney or by the existence of some potential common pathogenesis of cerebrovascular disease and renal disease [219-221]. And this still needs to be further explored.

#### 4.3.2 The role of the cerebral lymphatic system in bridging the gap between renal disease and cognitive dysfunction

End-stage renal disease is usually associated with cognitive dysfunction, typically manifested by inattention, impaired inhibitory control, memory loss, language, visuospatial performance and executive dysfunction, and sleep disturbances [222]. The cerebral lymphatic system is also an important factor mediating the onset of cognitive dysfunction due to kidney disease.

Current studies on chronic kidney disease (CKD) have shown that the accumulation of uremic toxins in the brain is an important cause of cognitive dysfunction in patients [206]. On the one hand, patients with chronic kidney disease (CKD) may induce anemia due to reduced erythropoietin production, which causes a compensatory increase in cerebral blood flow. This leads to an increased distribution of uremic toxins in the body to the brain and eventually to cognitive dysfunction. On the other hand, reduced erythropoietin production in CKD patients may lead to a decrease in hemoglobin content to some extent. This may cause cerebral hypoxia, which may likewise induce cognitive dysfunction [223]. A study by Chang Min Heo and his team showed that patients with chronic kidney disease already have dysfunction of the glymphatic system in the early stages of the disease [222, 224]. This is mainly due to two factors. On the one hand, astrocytes may be damaged or apoptotic due to the inflammatory response and oxidative stress triggered by the accumulation of uremic toxins in the brain [225-227]. On the other hand, changes in blood osmolarity in uremic patients may lead to altered AQP-4 expression [228].

Neurological complications induced by uremic toxin accumulation in the brain of patients with acute kidney injury (AKI) are usually more severe and develop faster than in patients with CKD [229, 230]. This may be mainly related to the development of pro-inflammatory responses, the release of pro-inflammatory cytokines, and alterations in the blood-brain barrier [231-233]. AKI is able to induce damage and inflammatory responses in the brain. It has been shown that the number of cells presenting GFAP-positive in the CA1 region of the hippocampus is significantly elevated after ischemia-reperfusion injury of the kidney. This predicts a massive proliferation of astrocytes [234, 235]. And as an important component in the cerebral lymphatic system, the proliferation of astrocytes may affect the normal function of the glymphatic system to some extent. The specific mechanism remains to be further explored.

## 5. Potential implications for disease pathogenesis and therapeutic approaches

### 5.1 Potential targets for the diagnosis and treatment of liver disease

#### 5.1.1 Hepatic encephalopathy

There is still a lack of specific indicators for the diagnosis of hepatic encephalopathy. However, Hajime Hirase and his team have done this by linking the gene for fluorescent protein to albumin and injecting it into C57BL/6JRj mice via a transgenic virus. They found that this induced the liver to produce modified albumin, which eventually produced a fluorescent tracer in the blood [236]. Since the liver can also produce lipoproteins, participate in processes such as glucose metabolism [237] and urea synthesis [238]. And these substances may also be potential targets for being modified. This may provide a novel idea for monitoring the progression of brain diseases in the future. However, it is worth thinking about the fact that the liver function of patients with hepatic encephalopathy has become severely impaired, resulting in its ability to synthesize proteins significantly diminished. Then, in order to achieve clear and precise tracing in different application scenarios, we should further determine the specific amount of fluorescent protein that should be induced in the liver and how to achieve this goal. This remains to be further explored.

Currently, the main clinical approach is to reduce blood ammonia concentrations by targeting the intestine and thus treating hepatic encephalopathy. However, this regimen has some limitations due to its long duration of action [189]. Therefore, considering that patients with hepatic encephalopathy already have a large accumulation of NH_3_ in the brain and the detoxification function of the liver is significantly reduced, accelerating the excretion of NH_3_ and other metabolic wastes in the brain of patients with hepatic encephalopathy may be another effective treatment for this disease. This may be achieved, for example, by promoting the production of new lymphatic vessels and increasing the flow of fluid. Vascular endothelial growth factor-C (VEGF-C) plays an important role in the formation of meningeal lymphatic vessels [7]. Its binding to VEGFR3 promotes the proliferation of lymphatic vessel endothelial cells and vascular endothelial cells, which in turn induces the generation of new lymphatic vessels and new blood vessels [204, 239]. For the brain, VEGF-C not only alleviates the neuroinflammatory symptoms in the mice with hepatic encephalopathy by increasing the drainage efficiency of the cerebral lymphatic system, but also suppresses microglia-induced inflammatory responses by upregulating Sik-1 expression and inhibiting the NF-kB signaling pathway [13, 240]. Therefore, we hypothesized that exogenous injection of VEGF-C might effectively reduce the accumulation of metabolic wastes in the brain and improve the symptoms of patients with hepatic encephalopathy by promoting the production of meningeal lymphatics and cerebral microvasculature. This has some promising applications. Meanwhile, for the liver, we speculate that upregulating VEGF-C in the liver may help establish effective collateral circulation in the liver by promoting hepatic angiogenesis. On the one hand, this could alleviate the symptoms of portal hypertension and liver ischemia and hypoxia in patients with cirrhosis. On the other hand, good collateral circulation could enable partial metabolism of NH_3_ in the liver and thus effectively relieve the neurological symptoms in patients with hepatic encephalopathy. However, the safety and efficacy of this potential treatment method still need to be explored and confirmed by numerous studies.

Aquaporin 4 (AQP-4) is the major water channel protein in the mammalian central nervous system. It is mainly found in the peduncle terminals of astrocytes and is important for maintaining normal water homeostasis in the CNS [165, 241, 242]. It also serves as a key hub for the glymphatic system to perform normal physiological functions and is closely related to fluid flow and substance exchange between the perivascular spaces and interstitial fluid [112]. On the one hand, enhanced AQP-4 expression can promote drainage of the glymphatic system, reduce the accumulation of metabolic waste in the brain, and thus improve the neurological symptoms in patients with hepatic encephalopathy [243]. But on the other hand, increased AQP-4 expression may also induce the formation of brain edema [244, 245]. From this perspective, down-regulation of AQP-4 expression could reduce brain edema and produce therapeutic effects in patients with hepatic encephalopathy complicated by brain edema. Therefore, it is important to find the balance between the two roles of AQP-4 in exerting enhanced lymphatic drainage and reducing cerebral edema. This still needs to be further explored.

#### 5.1.2 Brain metastasis from hepatocellular carcinoma

##### 5.1.2.1 Diagnosis

In addition to clarifying the potential mechanism of brain metastasis in hepatocellular carcinoma, the search for its early detection indicators has a key role. The most commonly used serological marker for diagnosing hepatocellular carcinoma in clinical practice is AFP. However, due to its lack of specificity, it is not possible to identify benign and malignant lesions in the liver by this indicator in some cases [246]. Therefore, it is clinically important to find specific diagnostic indicators for this disease. At present, the diagnosis of brain metastasis from hepatocellular carcinoma and its progression are mainly confirmed by clinical symptoms and imaging examinations. Patients with brain metastasis from hepatocellular carcinoma often have various clinical manifestations, such as headache, vomiting, hemianopia, disorientation, aphasia, hemiparesis, ataxia, mental deterioration, etc. Image examinations such as CT and MRI show tumors in the brain with edema. Laboratory tests often show significantly elevated levels of AFP, AFP- L3 and DCP in these patients [247].

In addition, Song et al. found that patients with esophageal cancer with positive expression of CCR7 mRNA and/or VEGF-C mRNA had a significantly higher recurrence rate of lymphatic metastases than those without expression of CCR7 mRNA and VEGF-C mRNA [248]. This suggests that VEGF-C and CCR7 may be reliable molecular indicators to mark lymphatic metastasis of tumors. Jianjun Zhao's team also found that serum levels of VEGF were higher in patients with hepatocellular carcinoma that had metastasized than in those without metastasis [249]. Therefore, VEGF-C may have some diagnostic value for brain metastasis from hepatocellular carcinoma. When patients develop brain metastasis from hepatocellular carcinoma, do the levels of VEGF-C and CCR7 in cerebrospinal fluid also change and become our molecular indicators to detect brain metastasis? Can the combination and indicators such as VEGF-C, CCR7 and tumor markers improve the accuracy of diagnosis? These questions remain to be further investigated.

##### 5.1.2.2 Potential therapeutic targets

For patients with brain metastasis from hepatocellular carcinoma, although treatment modalities such as surgical resection, whole brain radiation therapy, and stereotactic radiation therapy have shown some therapeutic effects in some studies, the survival of patients with brain metastases from hepatocellular carcinoma is still not long at present [250-256]. Therefore, it is important to find potential therapeutic targets for brain metastasis from hepatocellular carcinoma. The current treatment of brain tumors mainly relies on radiotherapy and chemotherapy. However, the existence of multiple factors has led to the treatment effect of brain tumor has been unsatisfactory. On the one hand, due to the existence of the blood-brain barrier, the entry of therapeutic drugs into the central nervous system is hindered, which can affect the efficacy to a certain extent. On the other hand, in the central nervous system, the T cell-mediated immune response to tumor antigens is very limited, which can easily lead to uncontrolled tumor growth and thus worsen the disease. Currently, the emerging cancer therapy of "immune checkpoint blockade" has received a lot of attention and has been applied in the treatment of breast cancer [257], hepatocellular carcinoma [258] and other types of tumors. Recent findings suggest that vascular endothelial growth factor C (VEGF-C) may enhance immune checkpoint blockade, thereby enhancing the body's immune response to tumors in the brain as well as other immune-exempt sites [259-263]. CCL19 or CCL21 binding to CCR7 activates signaling pathways in tumor cells, including PI3/Akt [264-266], MAPK/ERK [267-269], and Jak/STAT [269, 270], thereby enhancing VEGF-C expression. It has been shown that VEGF-C is an important factor in the early development of lymphatic vessels [271]. It has been shown that ectopic expression of VEGF-C may enhance T cell-mediated antitumor responses by affecting the function of meningeal lymphatic vessels. This includes enhanced initiation of anti-tumor T cells in deep cervical lymph nodes, migration of T cells to tumors, rapid clearance of brain tumors, and establishment of long-lasting anti-tumor immune memory [272]. Eric Song and his team induced high expression of VEGF-C in CSF by injecting VEGF-C mRNA constructs into the large pool of mice, which in turn enhanced endogenous immune responses [261]. This confirms, to some extent, that increased VEGF-C expression levels can play a positive role in the treatment of brain tumor. The introduction of exogenous VEGF-C as a therapeutic option has some feasibility. However, the combination of local injection of VEGF-C and immune checkpoint inhibitors is not perfect at present, and a lot of basic and clinical studies are still needed to prove its effectiveness and safety [273].

However, the act of upregulating VEGF-C also has a double-edged sword effect [274]. In terms of its positive significance, numerous studies have shown that VEGF-C can promote the priming of cancer cell antigens, enhance the initiation of antitumor T cells in deep cervical lymph nodes, the migration of T cells to tumors, and the establishment of long-lasting antitumor immune memory [261, 274-276]. However, in terms of its negative impact, the ability of VEGF-C to promote the proliferation and expansion of meningeal lymphatic vessels may promote lymphatic metastasis of brain tumours. Mihaela Skobe et al., Yulong He et al. and Satoshi Hirakawa et al. all suggested that overexpression of VEGF-C could effectively promote lymphatic vasculature generation, which in turn promotes lymphatic metastasis of tumor cells [272, 277, 278]. The results of the study by N Khromova et al. similarly suggest that downregulation of VEGF-C can inhibit the growth and metastasis of tumours [279]. At the same time, we cannot ignore the background condition that tumor cells can evade immune surveillance by activating immune checkpoints. These factors expose the obvious drawbacks of the strategy of achieving therapeutic goals by simply increasing VEGF-C expression levels in vivo. In this regard, we may be able to inhibit the binding of VEGFR-2/VEGFR-3 to VEGF-C by using receptor antagonists of VEGFR-2/VEGFR-3, thereby inhibiting the production of blood vessels and lymphatic vessels. This may not only limit the metastatic pathway of the tumor, but also reduce the blood and oxygen supply to the tumor. Thus, tumor growth is limited to some extent [280]. By combining anti-lymphangiogenic therapy with anti-angiogenic therapy, it may provide a new therapeutic strategy for the treatment of advanced cancer, which deserves further investigation.

In conclusion, it is very important to grasp the balance between the positive and negative effects of rising VEGF-C levels. In the process of applying VEGF-C in the treatment of brain metastasis from hepatocellular carcinoma, the primary problem to be solved is to find the balance between its positive and negative effects, so as to achieve the best therapeutic effect. But this needs to be further investigated. This may also be another effective way to prevent brain metastasis from tumors.

Meanwhile, as a kind of nano-vesicles containing various contents such as nucleic acids, proteins and lipids, exosomes have low toxicity, low immunogenicity and good biocompatibility. Therefore, they are considered as a good carrier for targeted drug delivery [281-284]. The application of exosome-targeted drug delivery in cancer treatment is one of the research hotspots in the scientific community today, so what is its role in hepatocellular carcinoma? As the current first-line drug for the treatment of hepatocellular carcinoma, long-term application of sorafenib may produce certain side effects and drug resistance [285, 286]. And the discovery of exosomes has provided a new solution to this problem. It has been shown that miR-122 in exosomes produced by hepatocellular carcinoma may play an important role in improving drug resistance and inhibiting vascular regeneration [287-289]. By encapsulating miR-122 into exosomes secreted from adipose tissue-derived mesenchymal stem cells and targeting their expression to hepatocellular carcinoma cells, it was able to improve the sensitivity of the organism to chemotherapeutic drugs [290]. In addition, Wang et al. demonstrated that treatment of hepatocellular carcinoma cells with miR-744-rich exosomes similarly enhanced the sensitivity of the organism to chemotherapeutic drugs [291]. Thus, targeted delivery to hepatocellular carcinoma cells by loading mi-RNA into exosomes may become a new therapeutic modality for patients resistant to chemotherapeutic drugs. If we target delivery to the liver or brain of patients at risk of brain metastasis by loading monoclonal antibodies to VEGF-C into exosomes, thereby inhibiting lymphatic vessels and angiogenesis, could this effectively reduce the risk of brain metastasis from liver cancer? For patients with brain metastasis, can we load a miRNA or an effective drug into exosomes and target it to the brain through the cerebral lymphatic system? The above questions still need to be confirmed by further studies.

### 5.2 Possible therapeutic approaches based on gastrointestinal regulation of the cerebral lymphatic system

Treatment of brain disorders through the gut is a hot topic of current research. Based on the close relationship between the gastrointestinal tract and the cerebral lymphatic system, we may achieve modulation of the cerebral lymphatic system by interfering with the microorganisms in the gastrointestinal tract or with the help of the intestinal lymphatic system. Currently, the main therapeutic measures to regulate the intestinal flora include nanocapsules [292], photobiomodulation [293], electroacupuncture or acupuncture [294], diet [295], probiotics [296], antibiotics [297], fecal bacteria transplantation [298], etc.

#### 5.2.1 Nanovesicles

Currently, various types of cell-derived nanovesicles and synthetic nanodrug carriers offer new possibilities to deliver drugs for the treatment of cerebral diseases through the cerebral lymphatic system [299, 300]. Compared with intravenous drug delivery, oral nanoparticle delivery has more advantages [301]. On the one hand, oral nanoparticle administration is relatively fast and convenient. On the other hand, oral nanoparticle administration is mainly absorbed and delivered through the lymphatic system. This can effectively avoid the first-pass elimination by the liver, thus solving the problem of poor pharmacokinetics that exists with the administration of nanovesicles through intravenous injection [302]. In addition, nanovesicles have good designability [299]. Therefore, can we design nanovesicles targeting astrocytes to achieve targeted therapy for the cerebral lymphatic system [303, 304]? For example, Xunwei Lai found that miR-146a-5p-modified exosomes derived from human umbilical cord mesenchymal stem cells could target neurotoxic astrocytes and attenuate their activation [292]. And this remains to be further explored.

#### 5.2.2 Photobiomodulation (PBM)

Brain using transcranial and intranasal PBM therapy has been shown to be effective in reducing neuroinflammation and promoting neuronal repair and regeneration [305, 306]. It also significantly enhances the drainage of meningeal lymphatics [305-308]. The strong penetration of near infrared (NIR) allows it to reach deep into the skull and is non-invasive throughout the operation. This makes it a potential option that can be used to treat brain diseases such as neurodegenerative disorders [309, 310]. In addition, PBM has a direct effect on the gastrointestinal tract. On the one hand, PBM therapy targeting the abdominal region may also alter the gut microbiota and affect the central nervous system through the release of as yet unidentified circulating mediators [293]. On the other hand, by irradiating the abdominal region, PBM therapy may enhance the permeability of the mesenteric lymphatic vessels. This may help to enhance the intestinal absorption of oral nanovesicular drugs. By pairing nanoparticle engineering with biophotonics, we may be able to achieve better therapeutic benefits. However, the appropriate irradiation dose for PBM therapy still needs to be explored in further studies.

#### 5.2.3 Acupuncture or electro-acupuncture

Numerous studies have demonstrated the unique role of acupuncture or electroacupuncture in regulating the gastrointestinal flora [311]. Electroacupuncture and acupuncture can cause an increase in the relative abundance of Lactobacillidae and elevated bacteroidetes/ Firmicutes in the gastrointestinal flora [312-314]. And acupuncture or electroacupuncture may be based on the regulation of intestinal flora, thus having some therapeutic effects on some central nervous system diseases, such as Alzheimer's disease [313], Parkinson's [315], and vascular dementia [316]. Currently, Yang B et al. found that acupuncture targeting APP/PS1 mice was effective in reducing the relative abundance of Porphyrionaceae and Helicobacter pylori family [317]. Porphyrionaceae [318] and H. pylori [319] have been shown to be associated with Alzheimer's disease. In particular, Porphyromonas gingivalis, a member of the Porphyromonas family, has been shown to cause dysfunction of the cerebral lymphatic system [15]. The specific acupuncture points vary for different diseases, including Baihui (DU20) [320, 321], Hegu (LI4) [322, 323], and Zusanli (ST36) [324, 325]. The application of acupuncture or electroacupuncture in the middle of the treatment process has good prospects, but still needs to be further explored.

#### 5.2.4 Dietary Patterns

Dietary patterns are strongly associated with both gut microbes and the brain [326-328]. A high-fat, high-sugar diet affects both the relative abundance of gut microbes in female mice and leads to a reduced density of astrocytes in the hypothalamus [329-331]. By feeding triple transgenic 3xTg-AD (TG) female mice withcombination of dried nopal, soy, chia oil, and turmeric for 7 months, astrocyte activation was effectively reduced with attenuated neuroinflammation [295]. Interestingly, it was found that water channel proteins from corn, soy, spinach leaves, and tomatoes are homologous to AQP-4 on astrocytes in the human brain. Antibodies against aquaporins produced as a result of food may lead to autoimmune diseases under specific circumstances [332]. These all suggest that dietary patterns may have an impact on the cerebral lymphatic system. And special dietary patterns may be a new way to intervene in the cerebral lymphatic system.

#### 5.2.5 Probiotics, prebiotics or synbiotics

Probiotics, prebiotics and synbiotics are able to reduce astrocyte activation and neuroinflammatory responses by regulating intestinal flora. Ultimately, it may affect the function of the cerebral lymphatic system [130, 333-338]. Lactobacillus rhamnosus administration attenuates microglia and astrocyte activation [339]. Jing Lu et al. found that by giving Lactobacillus acidophilus and Bifidobacterium infantis (LB) to pregnant C57/BL6J female mice, it promoted brain development and reduced astrocyte activation in their offspring [340]. However, due to the slow action of probiotics on intestinal flora and their colonization in the intestine is transient [333, 341]. Therefore, combining probiotics with nanovesicles may be a better strategy [342].

#### 5.2.6 Antibiotics

Antibiotics can have some effects on the gastrointestinal flora [343]. Also, antibiotics can have some effects on the central nervous system [297, 344-348]. For example, combination antibiotic therapy was able to significantly affect the relative abundance of Lachnospiraceae in the gastrointestinal flora. However, it was also able to inhibit the activation of astrocytes [349, 350]. Therefore, we hypothesize that antibiotics may indirectly affect the CNS by affecting the gastrointestinal flora in addition to directly affecting it.

#### 5.2.7 Fecal Materia Medica Transplantation (FMT)

Fecal transplantation is a method to rapidly alter the intestinal flora. It is mainly achieved by transferring intestinal flora from a healthy donor to the patient [351]. Fecal transplantation can reduce astrocyte activation and attenuate neuroinflammation by inhibiting NF-κB signaling pathway [352, 353]. Therefore, considering the importance of astrocytes for maintaining the normal function of the cerebral lymphatic system, fecal transplantation may also be a new idea to intervene in the cerebral lymphatic system.

### 5.3 Potential targets for the diagnosis and treatment of kidney disease

#### 5.3.1 Diagnosis

Some metabolites in the central nervous system are first transported from the interior of the brain through the glymphatic system to the subarachnoid space and then drained to the peripheral lymphatic system through the meningeal lymphatic vessels. Eventually, metabolic wastes and toxins that are drained to the periphery via the cerebral lymphatic system are excreted through the kidney. Currently, tests for patients with cognitive dysfunction or cerebrovascular disease caused by renal disease are often not specific. In contrast, fluorescent protein tracer technique can help us to understand the basic situation of fluid flow in the body such as blood circulation and lymphatic circulation. And it can help us diagnose diseases by detecting changes in fluid flow [236]. Since both kidney and brain are high blood flow organs, there are more possible scenarios for the application of fluorescent protein tracing technique in the diagnosis of brain and kidney diseases. A recent study conducted by Xiaowen Wang et al. used a single intraperitoneal injection or intravenous injection of adeno-associated viral vector (AAV) to induce the hepatocytes to express fluorescent protein-tagged albumin. This enables stable labeling of blood for more than 3 months and can be progressively metabolized without long-term accumulation in the body [236]. Interestingly, there is a large observable fluid flow in the cerebral lymphatic system, circulatory system, and urinary system. Therefore, by monitoring the entire process of fluorescently labeled metabolic waste moving with fluid flow in the body, we can promptly detect dysfunctions in organs such as the brain and kidney, thus helping us to update our knowledge of many diseases and provide new diagnostic and treatment options.

#### 5.3.2 Treatment

##### 5.3.2.1 Current treatment status

The current main treatments for kidney diseases include dialysis, kidney transplantation, oral administration of activated charcoal with adsorptive properties, and hemoperfusion. Their main purpose is to remove metabolic waste to alleviate the cognitive dysfunction and cerebrovascular damage caused by them [354-357].

###### 5.3.2.1.1 Dialysis

Dialysis is one of the main treatments for patients with end-stage renal disease (ESRD). After receiving hemodialysis treatment, the concentration of toxic proteins such as Aβ in the blood and cerebrospinal fluid of patients decreased significantly. This suggests that hemodialysis may inhibit the progression of neurodegenerative diseases [218, 358]. And the effect of hemodialysis on the cognitive function of ESRD patients may have a phase change. At the very beginning patients have a dramatic decline in cognitive function. Subsequently, cognitive function improves significantly in ESRD patients early in treatment, but deteriorates in ESRD patients in the intertreatment period [358, 359]. The dramatic decline in cognitive function during dialysis may be related to recurrent cerebral ischemia caused by blood pressure and hemodynamic instability during hemodialysis treatment [359-362]. In addition, patients often have concurrent cerebral edema during hemodialysis, which may also contribute to the dramatic decline in cognitive function during hemodialysis. During rapid dialysis, urea accumulates excessively among astrocytes due to the reduced expression of UT-B on astrocytes in CKD patients. At the same time, the flow of fluid into the brain interstitium increases due to the increased expression of AQP-4. Ultimately, the two together lead to the development of cerebral edema [363]. Therefore, the glymphatic system is important in the development and progression of cerebral edema in CKD patients receiving hemodialysis. In addition, newer methods of hemodialysis such as nocturnal hemodialysis or the use of cooled dialysate can better reduce cognitive dysfunction in patients compared to traditional hemodialysis methods [223]. It is important to note that different dialysis modalities can lead to changes in the incidence of cognitive dysfunction and cerebrovascular disease in ESRD patients. ESRD patients receiving peritoneal dialysis have significantly better cognitive function than those receiving hemodialysis [364]. As a continuous and gentle form of dialysis, peritoneal dialysis is effective in removing metabolic waste without causing dramatic fluctuations in blood pressure and hemodynamics. Therefore, peritoneal dialysis is more conducive to the recovery of cognitive function in ESRD patients. However, it is important to note that peritoneal dialysis improves cognitive function significantly slower than hemodialysis. Therefore, the mode of dialysis needs to be chosen according to the actual situation [365].

In a long-term perspective, dialysis can reduce the likelihood of cerebrovascular disease by reducing endothelial damage due to its filtering and removal of metabolic waste. However, in the short term, dialysis may induce the development of cerebrovascular disease. According to studies, the incidence of stroke is significantly higher in dialysis patients than in the healthy population and is dominated by hemorrhagic strokes. This may be due to the application of antithrombotic drugs during hemodialysis [218]. In addition, the vulnerability to stroke may be related to the dramatic changes in hemodynamics during dialysis and secondary factors such as blood pressure, vascular amyloidosis, and vascular calcification [215]. Therefore, maintaining hemodynamic and blood pressure stability during hemodialysis and applying antithrombotic drugs rationally are particularly important to prevent the occurrence of cerebrovascular diseases. In addition, peritoneal dialysis has a greater advantage in safety compared to hemodialysis because antithrombotic drugs are not required during the process [365].

###### 5.3.2.1.2 Kidney transplantation

Kidney transplantation is the ideal treatment for patients with chronic kidney disease. Studies have shown that kidney transplantation can significantly improve cognitive function in patients. During the three months after kidney transplantation, the cognitive function of transplant patients does not differ from that of dialysis patients, but the concentration of uremic toxins in the blood of transplant patients changes significantly [355]. Three months after the kidney transplantation, the cognitive function of transplant patients begins to improve significantly [223]. In addition, the patient's health status can greatly affect the recovery of cognitive function after kidney transplantation. It has been noted that the cognitive function of patients with kidney transplantation within one year postoperatively does not show much difference depending on their health status. However, after one year postoperatively, cognitive function in patients with frail health status can show a significant decline [366].

###### 5.3.2.1.3 Oral adsorption of activated carbon and blood perfusion

Both oral adsorptive activated charcoal and hemoperfusion apply adsorbents to adsorb uremic toxins produced by metabolism in the body and excrete them. The difference is that orally adsorbed activated charcoal such as AST-120 adsorbs uremic toxins and their precursors produced by microbial metabolism in the gastrointestinal tract and causes their excretion in the feces [367]. AST-120 reduces serum IS levels in a dose-dependent manner, and administration of AST-120 reduces the accumulation of IS and oxidative stress in rats with chronic kidney disease and subsequently improves endothelial dysfunction [367-369]. In contrast, hemoperfusion is the application of adsorbents to absorb small and medium molecular weight metabolic wastes and with protein-bound uremic toxins (PBUTs) in the purification system outside the body [370]. Thus, oral administration of adsorptive activated charcoal and blood perfusion can alleviate to some extent the cognitive dysfunction and cerebrovascular disease caused by the accumulation of uremic toxins.

##### 5.3.2.2 Potential therapeutic approaches based on the cerebral lymphatic system

###### 5.3.2.2.1 Treatment of cognitive dysfunction caused by kidney diseases based on cerebral lymphatic system

Current thinking on this aspect is focused on two perspectives by increasing the efficiency and working time of the cerebral lymphatic system. On the one hand, current studies have shown that AQP-4 facilitators, such as TGN-073, significantly increase the transport of water from cortical areas to the perivascular spaces, which in turn promotes the renewal of interstitial fluid and ultimately leads to enhanced clearance of the glymphatic system [243, 371]. However, at the same time, the use of TGN-073 may increase the incidence of cerebral edema during hemodialysis. And this remains to be further studied. It has been shown that MSC-derived exosomes can effectively inhibit the proliferation and inflammatory response of reactive astrocytes [372]. Therefore, we speculate that MSC-derived exosomes may enhance the clearance function of glymphatic system by repairing damaged astrocytes, and thus improve the cognitive dysfunction of the organism. This remains to be further explored by researchers. On the other hand, the clearance function of the glymphatic system is mainly active in the 3-4 phase of NREM [9]. And patients with chronic kidney disease are often accompanied by sleep disorders [373]. Therefore, by relieving patients' sleep disorders, i.e., prolonging stages 3-4 of NREM, it may provide new ideas for treating cognitive dysfunction in patients with chronic kidney disease.

###### 5.3.2.2.2 Treatment of cognitive dysfunction due to kidney disease based on renal excretory function

Most neurodegenerative diseases usually manifest as cognitive dysfunction, and their pathogenesis is related to protein deposition with altered physicochemical properties [374]. Promoting the clearance of deposited proteins in the brain is important to prevent neurodegenerative diseases or to slow down the process of neurodegenerative diseases. The kidney, as the main organ for excreting metabolic wastes and toxins from the body [206], also plays an important role in the development of neurodegenerative diseases. It has been shown that the kidney has a strong ability to remove β-amyloid and plays a key role in maintaining normal levels of β-amyloid in the blood and brain. Furosemide was able to improve cognitive dysfunction in AD mice by enhancing renal clearance of β-amyloid [128]. Therefore, we speculate that the combination of AQP-4 promoter TGN-073 and diuretics (e.g., furosemide) may both promote the glymphatic system for the clearance of metabolic waste in the brain and enhance the clearance function of the kidney. And this needs to be further validated. In addition, mesenchymal stem cells (MSCs), as a multifunctional cell population with self-renewal and differentiation, have potential therapeutic effects in various renal diseases. MSCs can specifically localize to the injured region of the kidney and exert therapeutic significance by secreting trophic factors or delivering subcellular structures [375]. Numerous studies have shown that MSCs therapy can improve renal function through multiple mechanisms, including anti-inflammatory, anti-apoptotic, anti-fibrotic, and pro-angiogenic. For example, cell therapy with MSCs has been shown to significantly improve the symptoms of AKI patients [376]. Therefore, the improvement of renal excretory function may contribute to the drainage of the glymphatic system and the elimination of metabolic waste in the brain.

These findings suggest that transport between the brain and periphery is important for the clearance of metabolic waste and the maintenance of brain homeostasis. Reduced clearance by the peripheral system may impede circulatory processes and lead to the accumulation of pathological proteins in the central nervous system. Therefore, enhanced peripheral waste removal may alleviate the pathology associated with Aβ in the brain [127]. It may provide a new therapeutic approach for neurodegenerative diseases.

From the present perspective, the cerebral lymphatic system acts as a bridge between the brain and the peripheral system, allowing us to fully understand and appreciate the pathogenesis and potential therapeutic directions of neurodegenerative diseases from a systemic perspective. There are relatively few studies revealing the specific mechanism of the connection between the cerebral lymphatic system and peripheral tissues and organs, which may become a potential development direction and hot spot in this field in the future.

## 6. Conclusions and future directions

In this review, we first describe the structural components and functional properties of the cerebral lymphatic system. Secondly, we review the evidence for the involvement of the cerebral lymphatic system in some peripheral system diseases. Finally, we describe the role of the cerebral lymphatic system as a potential bridge between the central nervous system and the peripheral system and provide new ideas for the diagnosis and treatment of diseases of the peripheral system.

As a special lymphatic system existing in the central nervous system, the cerebral lymphatic system differs considerably from the peripheral lymphatic system known to us. This is mainly due to the unique flow patterns and pathways of the glymphatic system. This is mainly due to the unique way in which the glymphatic system flows and the pathways through which it flows. Further research on the structure and function of the cerebral lymphatic system is essential for a better understanding of the brain and for exploring further clinical applications. At this stage, there are a number of issues that need to be addressed in the study of the cerebral lymphatic system. First of all, we need to clarify whether there is a certain degree of limitation on the size and type of molecules that pass through the cerebral lymphatic system. This mainly refers to the fluid exchange that occurs at the AQP-4 of the astrocyte endfeet. In the second place, we need to clarify more about the specific locations and mechanisms of solute and immune cell exchange that occur between the meningeal lymphatic vessels and the cerebrospinal fluid at different sites. This will assist us to clarify the specific sites and methods of operation for therapeutic regimens which target the meningeal lymphatic vessels. Furthermore, the specific processes and mechanisms of interaction between the cerebral lymphatic system and the peripheral lymphatic system are still unclear.

As a bridge between the central nervous system and the peripheral system, the cerebral lymphatic system has also attracted our attention to the peripheral system at the same time. As more and more attention is now being paid to the impact of the peripheral system on central nervous system diseases, we have come to realise that the traditional concept of treating the central nervous system separately from the rest of the body is not justified. Diseases of the central nervous system should be treated as systemic diseases. Peripheral organs, tissues and cells such as the liver, kidneys, gastrointestinal tract, skin and mononuclear cells have gradually become new breakthroughs in the treatment of central system diseases. At the same time, the indirect aim of treating peripheral system diseases by improving the health of the central nervous system is also a highly viable potential idea. However, it is important to note that the peripheral system is closely linked to the immune function of the central nervous system. Intervention of peripheral immune cells for dual immunotherapy of the periphery and the centre is a promising area of research, but its efficacy remains to be investigated.

Venous drainage is the main drainage route for metabolic waste in most organs of the body. Thus, tests and analyses of blood in the veins are used as the main diagnostic and therapeutic target for various diseases. However, due to the presence of the blood-brain barrier, the main diagnostic and therapeutic target for central nervous system diseases have been replaced by the cerebrospinal fluid, which usually requires invasive procedures [377-379]. The cerebral lymphatic system, which has recently been shown to be closely associated with the flow of cerebrospinal fluid, has great potential to become a diagnostic and therapeutic target and may play a revolutionary role in the advancement of a non-invasive approach to CNS diagnosis and treatment. At the diagnostic level, the previously proposed method of assessing the flow of the glymphatic system by intrathecal injection of gadolinium contrast agent is an invasive procedure that is only suitable for a limited number of patients with clear clinical indications. Moreover, the tendency of gadolinium accumulating in the central nervous system renders this method as a potential health risk for the patients [380]. Therefore, non-invasive imaging methods that do not require contrast agents are preferred by clinicians. It has been shown that the "Diffusion Tensor Image-Analysis aLong the Perivascular Space (DTI-ALPS)" methods based on non-invasive MRI can effectively detect brain lymphatic system dysfunction and quantify the activity of fluid flow in patients with idiopathic normal pressure hydrocephalus [381]. This has amply demonstrated the feasibility of a non-invasive, effective and standardised clinical imaging method for the cerebral lymphatic system and has inspired further exploration [382-384]. Currently, various imaging modalities targeting the glymphatic system have shown relatively obvious drawbacks. The observation range of two-photon laser scanning microscopy is relatively limited. MRI coupled with intrathecal gadolinium-based contrast agents has poor resolution for delicate anatomical structures [3, 9, 40, 385-387]. Thus, the developing whole brain imaging techniques may enable us to systematically visualize the intact cerebral lymphatic system within the CNS and thus further reveal the specific pathways of fluid flow in the CNS. In the near future, this may become an indispensable basis for the clinical assessment of the function of the cerebral lymphatic system. In terms of treatment, non-invasive physical therapies are gaining more and more attention, such as photobiomodulation (PBM) therapy [388]. In addition, the use of nanoparticle engineering in conjunction with biophotonics during therapeutic procedures may provide better therapeutic benefits due to the high blood-brain barrier crossing properties and low toxicity of nanoparticles [389].

Current treatment options for central nervous system disorders usually consider stem cells as the primary treatment and rarely consider targeting immune cells. This is partly influenced by the traditional view of immune isolation of the brain. On the other hand, there is still a slight gap in research on the types of immune cells in the brain, their distribution, and when and how they function. However, as our understanding of the brain's lymphatic system improves, brain immunotherapy may become an important treatment tool for us in the future. According to current research, the immune response in the brain may be a double-edged sword. In the acute phase, the rapid and powerful immune response produces damage to brain tissue that is difficult to ignore. In the chronic phase, the immune response is able to remove potential pathogenic metabolic wastes from the brain and thus exerts a protective effect. This contributes to the recovery of damaged tissue. Therefore, in the future, we must select individualised treatment plans for different periods of the diseases with full consideration of the dual role of the immune system.

Efficient, low-damage drug delivery systems for the brain are currently receiving increasing attention. On the one hand, many attempts have been made to efficiently deliver drugs from the peripheral system into the central nervous system, such as improving the permeability of the blood-brain barrier, using lipid-soluble drugs, or employing emerging nanoparticle technologies. Rui Liu and his team systematically tracked nonreactive gold nanoclusters delivered to the brain and found that they could cross the blood-brain barrier through active transport by exosomes. At the same time, they can also exit the brain via the glymphatic system. The discovery of the cerebral lymphatic system may mean that we can no longer demand that the drugs cross the blood-brain barrier, but only that the drugs with the most suitable functional characteristics for our therapeutic needs are considered. On the other hand, by exploiting the ability of the glymphatic system to penetrate deeply into the brain parenchyma, we may be able to explore new delivery options for intrathecal or intracerebroventricular drug injections on this basis. Such innovative CNS drug delivery routes could be of great practical interest and research value. For example, it has recently been shown that antagonism of the α3 GABAA receptor enhances the activity of delta waves, thereby promoting deep sleep of animals [389]. So, could we later antagonise this receptor by feeding specific drugs into the cerebral lymphatic system to improve sleep quality or even meet the criteria for clinical sleep treatment? Further research in this area is likely to answer many similar questions.

The cerebral lymphatic system is expected to play a crucial role in the current development of treatments for various diseases, and there is growing evidence to support its potential as a diagnostic and therapeutic target for the prevention and treatment of diseases of the central nervous system and peripheral system. This remains to be further investigated and explored. In conclusion, this review contributes to a better understanding of the structure and function of the cerebral lymphatic system. As a bridge between the central nervous system and the peripheral system, it has enormous potential for new directions in the diagnosis and treatment of various diseases. We hope that this review will be helpful and enlightening for the study of the cerebral lymphatic system.
